# Comparative Survival and the Cold-Induced Gene Expression of Pathogenic and Nonpathogenic *Vibrio Parahaemolyticus* from Tropical Eastern Oysters during Cold Storage

**DOI:** 10.3390/ijerph17061836

**Published:** 2020-03-12

**Authors:** Francisco Alarcón Elvira, Violeta T. Pardío Sedas, David Martínez Herrera, Rodolfo Quintana Castro, Rosa María Oliart Ros, Karla López Hernández, Argel Flores Primo, Karen Ramírez Elvira

**Affiliations:** 1 Facultad de Medicina Veterinaria y Zootecnia, Universidad Veracruzana, Av. Miguel Ángel de Quevedo s/n esq. Yáñez, Col. Unidad Veracruzana, Veracruz, Ver. CP 91710, Mexico; ibq_alarcon@hotmail.com (F.A.E.); dmartinez@uv.mx (D.M.H.); karlop_80@yahoo.com.mx (K.L.H.); mopri02@yahoo.com.mx (A.F.P.); krelvira16@hotmail.com (K.R.E.); 2 Facultad de Bioanálisis, Universidad Veracruzana, Calle Iturbide s/n, Col. Centro, Veracruz, Ver. CP 91700, Mexico; rodolfoquint@yahoo.com; 3 Unidad de Investigación y Desarrollo en Alimentos, Instituto Tecnológico de Veracruz, Av. Miguel A. de Quevedo 2779, Veracruz, Ver. 91897, Mexico; roliart@itver.edu.mx

**Keywords:** *Vibrio parahaemolyticus*, postharvest refrigeration, cold shock, growth kinetics, gene expression

## Abstract

Expression of the regulatory stress *rpoS* gene controls the transcription of *cspA* genes, which are involved in survival and adaptation to low temperatures. The purpose of this study was to assess the growth kinetics of naturally occurring *V*. *parahaemolyticus* in shellstock oysters and in vitro and the cold-shock-induced expression of the *rpoS* and *cspA* gene response in vitro during postharvest refrigeration. Naturally contaminated eastern oysters (*Crassostrea virginica*) and pathogenic (*Vp-tdh*) and nonpathogenic (*Vp-tlh*) isolates were stored at 7 ± 1 °C for 168 h and 216 h, respectively. The regulatory stress (*rpos*) and cold-shock (*cspA*) gene expressions were determined by reverse transcription PCR. At 24 h, the (*Vp-tdh*) strain grew faster (*p* < 0.05) than the (*Vp-tlh*) strain in oysters (λ = 0.33, 0.39, respectively) and in vitro (λ = 0.89, 37.65, respectively), indicating a better adaptation to cold shock for the (*Vp-tdh*) strain in live oysters and in vitro. At 24 h, the (*Vp*-*tdh*) strain *rpoS* and *cspA* gene expressions were upregulated by 1.9 and 2.3-fold, respectively, but the (*Vp-tlh)* strain *rpoS* and *cspA* gene expressions were repressed and upregulated by −0.024 and 1.9-fold, respectively. The *V*. *parahaemolyticus* strains that were isolated from tropical oysters have adaptive expression changes to survive and grow at 7 °C, according to their virulence.

## 1. Introduction

*Vibrio parahaemolyticus* is a gram-negative halophilic bacterium that commonly inhabits marine and estuarine waters worldwide. Infections caused by *V. parahaemolyticus* have increased in different parts of the world in recent years [1]. This pathogen is the leading cause of gastroenteritis associated with the consumption of raw, undercooked, or poorly marinated seafood, including fish, crustaceans, and, particularly, oysters [2]. Thermolabile hemolysin (TLH), encoded by the *tlh* gene, is used to identify both clinical and environmental strains of *V. parahaemolyticus* [3]. The main factors of *V. parahaemolyticus* virulence are thermostable direct hemolysin (TDH), encoded by the *tdh* gene, and thermostable-related hemolysin (TRH), encoded by the *trh* gene. However, the complete mechanism of pathogenesis remains unclear. These genes have typically been reported in low percentages (1% to 2%) of nonclinical isolates and about 10% of clinical strains do not contain *tdh* and/or *trh* [4]. However, we have reported a significantly higher frequency of the *tlh+/tdh*+ gene during winter (15.4%) in *V. parahaemolyticus* isolates from eastern oysters (*Crassotrea virginica*) harvested in the Mandinga Lagoon System (MLS) [5]. Another study in the state of Sinaloa, Mexico found that 71.7% of environmental strains of *V. parahaemolyticus* had the *tdh* gene [6]. Environmental isolates lacking *tdh* and/or *trh* are also highly cytotoxic to human gastrointestinal cells. Even in the absence of these hemolysins, *V. parahaemolyticus* remains pathogenic, indicating that other virulence factors exist [4]. The more infectious pandemic serotype O3:K6 contains the *orf8* gene, a filamentous phage that is associated with pandemic strains of *V. parahaemolyticus*. The *orf8* gene is believed to encode an adherence protein that increases the ability of *V. parahaemolyticus* to adhere to host intestinal cells or to the surfaces of marine plankton. The occurrence of *orf8* genes in foodborne *V. parahaemolyticus* strains has been reported and considered as an additional virulence factor [7]. In México, between 2004 and 2010, recurrent cases of gastroenteritis were reported in both South and North areas of the state of Sinaloa in Northwest Mexico, causing >79% of reported cases due to pandemic O3:K6 strains of *V. parahaemolyticus* [6]. In the state of Veracruz, 27 cases of *V. parahaemolyticus*-induced diarrhea were recorded in 2014, of which two were in the Port of Veracruz. In 2015, 27.70% of the state’s cases were reported in the metropolitan zone of Port of Veracruz-Boca del Río [8].

Refrigeration has been the most commonly used method for short-term storage to preserve quality and extend the shelf life of shellfish. Maintaining the cold chain from harvest through to consumption is critical for preserving freshness and quality as well as for ensuring the safety of shellfish products [9]. Nevertheless, the safety of oysters may be compromised because *V. parahaemolyticus* strains vary in their ability to survive and grow at low temperatures. It has been reported that the concentration of pathogenic *V. parahaemolyticus* (*trh*+) decreased (*p* < 0.05) faster than that of nonpathogenic (*tlh+*) in broth and oyster slurry at 10 °C [10]. In contrast, the density of *V. parahaemolyticus* nonpathogenic (*tlh+*) and pathogenic (*tdh+*, *trh*+, *tdh+/trh+*, and *tdh+/orf8+*) strains increased significantly by day 3 in eastern oysters (*C*. *virginica*) stored at 7 °C for 9 days [11]. It has been reported that during cold storage (3 °C), *V. parahaemolyticus* undergoes physiological stress [12]. This stress response involves changes in the gene expression and drastically alters the physical and biological parameters of a living cell [13]. To adapt to cold shock, the expression of *rpoS*, a regulatory gene, controls the transcription of specific genes, such as *cspA* [14]. It has been observed that the *cspA* gene, which encodes cold shock protein (CSP), showed a 30-fold upregulation of the transcriptional level at 10 °C, but below this temperature, bacterial growth was arrested; this outcome suggests that CspA could be the major cold shock protein in *V. parahaemolyticus* during low-temperature growth [15].

In Mexico, oysters are harvested extensively within the oyster-producing areas found along the Mexican Gulf Coast. The state of Veracruz is the primary oyster producer, with 42% (23,119 tons) of the annual average oyster harvest [16]. The Mandinga Lagoon System (MLS), located in the central region of the state of Veracruz, is one of the most important shellfish-producing estuarine lagoon systems along Mexico’s Gulf coast. The climate in this region is tropical [17]. The eastern oyster (*C. virginica*) is one of the most popular bivalve mollusks and is widely consumed in large quantities. Approximately 52,526 tons of oysters are consumed annually, with an annual per capita of 0.43 kg [16], and 85.9% of consumers eat them raw [18]. Oysters are sold live in the whole shell, fresh or refrigerated in sacks, shucked in the fresh form, or packaged and refrigerated in polyethylene bags. Oysters are sent packaged to nearby local markets, retail stores, restaurants, oyster bars, and purchased by street vendors mostly in the Metropolitan Area of Veracruz, which includes the municipalities of Veracruz, Boca del Río, and Alvarado [19].

According to the Mexican Norm NOM-242-SSA1-2009 [20], which provides guidelines for the sanitary control and commerce of shellfish in Mexico, shellstock oysters should be kept alive and adequately refrigerated at 7 °C for a maximum of 7 days to ensure safe consumption. Because of the importance of raw oysters in gastronomy and economics, their microbial safety is of major interest. Our previous studies have indicated that *V*. *parahaemolyticus* is capable of surviving during refrigeration [11]. This capability may be associated with the molecular mechanisms related to the expression of stress (*rpoS*) and cold-shock (*cspA*) genes. Despite its impact on public health and the oyster industry, the cold response of *V. parahaemolyticus* strains in tropical eastern oysters during low temperature storage has been scarcely studied. To better understand the *V. parahaemolyticus* response to refrigeration temperatures, the aim of our study was to assess the survival kinetics of nonpathogenic and pathogenic *V. parahaemolyticus* strains in live shellstock oysters, the in vitro survival kinetics of nonpathogenic and pathogenic *V. parahaemolyticus* strains isolated from the tropical eastern oyster (*C. virginica*), and the in vitro expression levels of the *rpoS* and *cspA* genes that are induced by cold shock at the shellstock temperature refrigeration storage conditions recommended under the Mexican Regulations.

## 2. Materials and Methods

### 2.1. Area of Study and Sample Collection

A total of 600 live oysters of medium legal size (7 to 8 cm long) (NMX-FF-001-SCFI-2011) [21] (SE, 2011) were harvested manually by divers from the banks of the Canal de Mandinga during the windy season and were immediately transported in coolers at 4 °C to the laboratory following the Mexican Norm of the Mexican Ministry of Health [20]. Dead or empty oysters were discarded (30–50 oysters) and the remaining oysters were scrubbed and rinsed under cold running tap water to remove debris and attached algae. Experiments on live animals were approved by the Bioethics and Animal Welfare Committee of Veterinary Faculty, confirming compliance with all requirements of Mexico (approval code 090116).

### 2.2. Survival under Cold Shock Conditions in Live Shellstock Oysters

After cleaning, 500 (250 oysters per replicate) naturally contaminated shellstock oysters were immediately stored in hermetic plastic boxes in the same laboratory chamber at the same time at 7 ± 1 °C for up to 7 days (168 h) to study the effect of temperature downshift (cold shock) on *V. parahaemolyticus* strains growth, imitating the time-of-harvest and time-of-refrigeration conditions in accordance with those currently mandated by the Mexican Minister of Health NOM-242-SSA1-2009 [20]. To identify and quantify the total and pathogenic density of *V. parahaemolyticus* strains, 40 oysters were shucked under aseptic conditions to attain 200 g of oyster sample (150 g of meat and 50 g of intravalvular fluid) and were analyzed at 0, 24, 72, 120, and 168 h in duplicate, following the MPN-PCR (Most Probable Number-Polymerase Chain Reaction) procedure described previously [5]. These time points were selected according to the storage period registered in the restaurants and cocktail bars surveyed [17]. This experiment was conducted three times during the season.

### 2.3. Isolation and Purification of V. Parahaemolyticus Strains from Oysters for an In Vitro Study

*V. parahaemolyticus* strains were isolated from the natural contaminated shellstock oyster samples during the former *in live* assay at 0 h. The isolation and purification of *V. parahaemolyticus* strains were carried out following the MPN-PCR (Most Probable Number-Polymerase Chain Reaction) procedure described previously [5]. First, 200 g of oyster sample (150 g of meat and 50 g of intravalvular fluid) were mixed with 200 mL phosphate-buffered saline (PBS) and blended for 120 s to make a 1:1 dilution. The shellfish homogenate was added to alkaline peptone water in a three-tube MPN dilution series prepared up to 1:10^4^ dilution according to the standard three-tube MPN procedure. The tubes were incubated at 35 °C for 24 h. After a 24 h incubation, one loopful from each positive broth tube was streaked onto plates containing CHROMagar™ Vibrio (CHROMagar Microbiology, Paris, France) and were incubated at 35 °C for 24 h. At least 20 well-grown mauve colonies of the presumptive *V. parahaemolyticus* from each inoculated CHROMagar plate were selected and inoculated in T1N8 agar and were incubated at 35 °C for 18 h for purification.

*V. parahaemolyticus* presumptive isolates were identified by biochemical characteristics using Kligler iron agar slants (KIA), lysine iron agar (LIA), motility-indole-ornithine medium (MIO), Moeller decarboxylase broth media, and arginine dihydrolase tests; pure control cultures (CAIM www.ciad.mx/caim) were subcultured onto nutrient agar (with 2% NaCl) and were tested as well. All agar media were BD Bioxon (Becton Dickinson de México S.A. de C.V., México). Afterwards, the oxidase test (p-aminodimethylaniline) (Becton Dickinson, NJ, USA) was performed on growth from presumptively positive isolates, which were confirmed by PCR analysis as described by us previously [5]. The *16S* ribosomal *rRNA* gene of *V. parahaemolyticus* isolates were amplified by PCR and were sequenced at the Biotechnology Institute of the National Autonomous University of México (UNAM) [22,23]. The sequences obtained were analyzed and compared with sequences from GenBank using BLASTn by NCBI (http://blast.ncbi.nlm.nih.gov). Among the *V. parahaemolyticus*, 20 that were confirmed positive were attained from live oyster assay at time 0 h, then 10/20 were identified as *tlh+* of which 3/10 were identified as *tdh+*. The 16S ribosomal RNA sequencing of the purified strains indicated a high genetic similarity (99.5%) with the reference sequence of *Vibrio parahaemolyticus* strain TJA114 partial sequence (GenBank accession no. MK796103.1). Analysis with the Serial cloner 2.6 software identified the *cspAI* gene. The strains that were scored as positive were maintained and routinely grown on Trypticase soy agar (TSA) (BIOXON Becton Dickinson S.A de C.V., Mexico) at 35 °C until the in vitro study. The commercially available O3-Group and K6-Group antiserum kit (Hardy Diagnostics, CA, USA) was used for serological O3:K6 (pandemic clone) tying of the *V. parahaemolyticus tdh+* strains following the manufacturer’s instructions. The isolated positive pathogenic (*Vp-tdh*) strains were not O3:K6 (pandemic clone). Hence, the *V. parahaemolyticus tlh+* and *tdh+* positive strains isolated from live oyster assay at time 0 h were used for the in vitro study as no *trh*+, *tdh/trh*+, and *orf8*+ strains were isolated.

### 2.4. Survival under Cold Shock Conditions In Vitro

In order to understand the effect of temperature downshift on the growth kinetics of *V. parahaemolyticus* after cold shock and the cold-induced expression of the regulatory stress (*rpos*) and cold shock (*cspA*) genes, five *tlh+* and the three *tdh+* purified strains were studied in vitro during refrigerated storage by mimicking the refrigeration conditions of 7 °C for up to 216 h (9 days), two more days than the 7 days currently recommended by the NOM-242-SSA1-2009. For the in vitro assay, one loopful of each of the pathogenic and nonpathogenic strains were inoculated separately in 25 mL of tryptone soy broth (TSB) (BBL/Difco Laboratories, Sparks, MD, USA) supplemented with 3% NaCl at 35 °C for 24 h. After incubation, the diluted cultures reached a population of 10^2 to 10^3 log CFU/mL. Then, 50 µL of broth cultures were inoculated in 50 mL tryptone soy broth supplemented with 3% NaCl in 500 mL Erlenmeyer flasks and were incubated at 35 °C for 2 h. After the incubation period, the cultures were transferred to 7 ± 1 °C (cold shock) for 216 h (9 days). At various time intervals from initial growth to 216 h (0, 24, 72, 120, 168, and 216 h), the viable count was monitored by surface plating 1 mL of each bacterial suspension according to the serial dilution procedure following the Mexican Norm NOM-242-SSA1-2009 approved method [20]. After serial dilutions, the viable count was made on triplicate CHROMagar™ Vibrio plates inoculated and incubated at 35 °C for 24 h. To detect any injured *V. parahaemolyticus* cells after cold shock, 1 mL of each bacterial suspension of the appropriate dilution was plated onto nutrient agar (BIOXON Becton Dickinson) in triplicate and incubated at 30 °C for 48 h. As a control, a sample of cells in tryptone soy broth was used. The total number of colony forming units in cells suspensions were enumerated; however, no injured cells were detected. Simultaneously, 12 mL of each broth culture was centrifuged at 19,500 xg for 15 min at 4 °C to obtain a pellet. The pellet was subjected to the RNA extraction protocol and DNA complementary synthesis (cDNA) to amplify the regulatory stress (*rpos*) and cold-shock (*cspA*) genes by reverse transcription PCR (RT-PCR) as described below. All strains were analyzed in triplicate.

### 2.5. In Vitro Gene Expression

To determine the expression of the cold shock *cspA* and stress response *rpoS* genes, the RNA was halted and extracted from *V. parahaemolyticus* cells using TRIzol® Reagent (Invitrogen, Carlsbad, CA, USA) by mechanical disruption and purified following the procedure previously described [24]. The absence of DNAg contamination in the RNA samples was verified by PCR. The PCR primers that were used target the expressed *rpoS* and *cspA* genes (Table 1). Agarose gel electrophoresis of the PCR products confirmed that no DNAg contamination was observed. The RNA concentrations were measured in triplicate at 260 nm by using a NanoDrop ND 2000 spectrophotometer (Thermo Fisher Scientific, Waltham, MA, USA). Purity of the RNA extracted was determined as the A_260_/A_280_ ratio. Isolated RNA quality was verified by an average A_260_/A_280_ ratio of 1.90 and 1.89 for the RNA extracted from the nonpathogenic (*Vp*-*tlh*) and the pathogenic (*Vp*-*tdh*) strains, respectively. An A_260_/A_280_ ratio > 1.8 is usually considered an acceptable indicator of good RNA [25]. The RNA integrity was evaluated through an agarose gel and visualized with UV light after electrophoresis. The gene expression was tested by reverse transcription PCR (RT-PCR) amplification. The cDNA was generated using 4 μg of total RNA for each strain with the GoScript™ Reverse Transcription System Kit (Promega, Madison, WI, USA) according to the manufacturer’s instructions. RT-PCR reactions were carried out with a StepOne Real-Time PCR system (Applied Biosystems, Waltham, MA, USA) using 100 ng of cDNA for each reaction. Each reaction system consisted of 10 μL of IQ SYBR green SuperMix (Bio-Rad Laboratories, Inc., Hercules, CA, USA), 0.3 μL each of 300 μM forward and reverse primers, 8.4 μL of RNase-free water, and 1.0 μL of cDNA template. The RT-PCR in 20 μL reaction volume was performed as follows: 95 °C for 3 min, 95 °C for 15 s, and 60 °C for 60 s for 40 cycles. The melting curve analysis was performed after each RT-PCR reaction to confirm that only RT-PCR products were detected. PCR amplification was performed with 2 μL of the synthesized cDNA that was amplified from the RT reaction as a template and products were run on 1.5% agarose gel. The expression level of the housekeeping *pvuA* gene was used as a constitutive expression control. Primers targeting the housekeeping, cold shock, and stress response genes were used for PCR for all strains. Each PCR was conducted in triplicate. The primers that were used for the identification of *V. parahaemolyticus* and the regulatory, cold-shock, housekeeping, and 16S rRNA genes in *V parahaemolyticus* are described in Table 1.

### 2.6. Data Analysis

The expression data were obtained for the reference gene of the threshold cycle (Ct) values. The 2^−^^ΔΔCt^ method was used to calculate differences in the expression of *rpoS* and *cspA* genes during storage period, and the gene expression was normalized to the endogenous reference gene *pvuA*. The amplification efficiencies were calculated using the relative quantification software provided by StepOne™ Real Time PCR (Applied Biosystems). The data were log_2_ transformed and the fold change in gene expression was obtained for each gene using the reference expression level at 0 h [30]. Genes with an adjusted *p*-value ≤ 0.05 and a logarithmic fold change ≥1.5 were considered to be differentially induced, while genes with a logarithmic fold change ≤ −1.5 were considered to be downregulated. To evaluate the relative quantification of the normalized gene expression data of the stress regulatory gene (*rpoS*), the cold shock gene (*cspA*), and the log_10_ CFU/mL of the pathogenic and nonpathogenic genes of *V. parahaemolyticus* during the time of refrigerated storage, the data were evaluated using analysis of variance and Tukey’s test to detect differences based on storage time. The statistical analyses were performed using the statistical software *X*LSTAT 2019 software (Addinsoft™, New York, USA) with a significance level of *p* < 0.05.

A modified Gompertz model was fit to the experimental pathogenic and nonpathogenic *V. parahaemolyticus* (log_10_ CFU/mL) data obtained at 7 °C using Statistica 7.0 software (Statsoft, Palo Alto, CA, USA) to determine the lag time and specific growth rate. This model has been used to describe *V. parahaemolyticus* growth [10]:

(1) $$Y_{t}=\;N_{0\;}+\;A\;\times \;exp\;\left[-exp\;\left(2.718\;\times \;\frac{{µ}_{\max}}{A}\right)\;\times \;\left(\lambda -t\right)\;+\;1\right]$$

where $Y_{t}$ is the log counts (CFU g^−1^) at time *t*; $N_{0}$ is the initial level of bacteria (log CFU g^−1^); *A =* log_10_ *(N_max_/N_0_),* where *N_max_* represents growth from the inoculum to stationary phase; and the parameters exp, µ_max_, and λ represent *e* constant, maximum specific growth rate (h^−1^), and the lag time of the strain growth (h), respectively.

The effect of temperature on *Vp* growth was calculated with Equation (2):

*G* = ln2/*µ_max_* (2)

where *G* is the generation time (h) at 7 °C and µ_max_ is the maximum specific growth rate (h^−1^).

The goodness of fit of the modified model was evaluated using the coefficient of determination (*R*^2^) and the standard deviation of the residuals (*Syx*), which were calculated using Statistica software.

## 3. Results and Discussion

### 3.1. Effect of Cold Shock on Growth Kinetics of Nonpathogenic (Vp-tlh) and Pathogenic (Vp-tdh) V. Parahaemolyticus in Live Oysters

**Figure 1:**
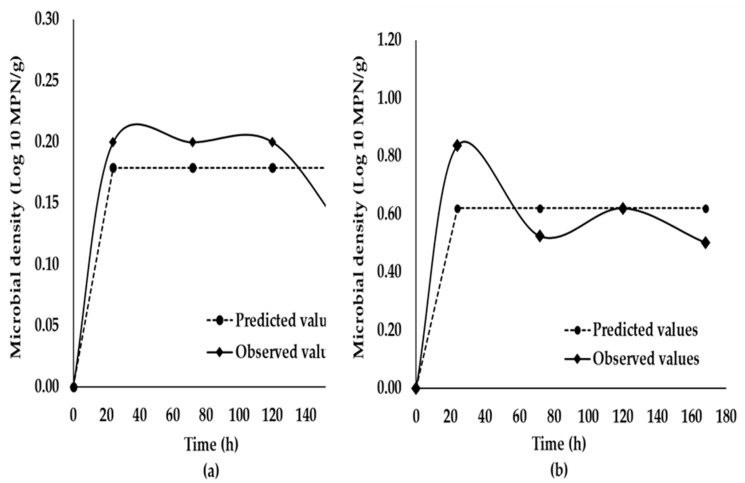
Comparison of the predicted and observed nonpathogenic *tlh*+ (**a**) and pathogenic *tlh+/tdh*+ (**b**) growth in live oysters (*Crassostrea virginica*) during refrigerated storage at 7 ± 1 °C for 180 h. Predicted growth values were fitted using Equation (1).

The average *V. parahaemolyticus tlh+* and *tdh+* numbers in natural contaminated oysters at 0 h were 2.84 and 0.56 log MPN/g, respectively. No *trh+*, *tdh/trh*+, and *orf8*+ levels were detected (0.15 log MPN/g) at 0 h or throughout the storage. Only (*Vp*-*tlh*) and (*Vp*-*tdh*) levels were detected throughout the storage period. Thus, the growth curves of (*Vp*-*tlh*) and (*Vp-tdh*) were fit from the experimental data and are presented in Figure 1a,b, respectively. The initial loads of (*Vp*-*tlh*) and (*Vp*-*tdh*) at time zero increased (*p* > 0.05) at 24 h but decreased (*p* > 0.05) at 168 h. The values for the kinetic growth parameters and performance statistics of the modified Gompertz model at 7 °C are shown in Table 2. The average *R*^2^ values of the model for the growth curves in live oysters were 0.8300 and 0.8100 for the (*Vp-tlh*) and the (*Vp*-*tdh*) strains, respectively. The predicted lag time value (λ) of the (*Vp-tlh*) strain was close to that of the (*Vp*-*tdh*) strain, indicating similar growth and adaptation of both strains to cold shock. The maximum specific growth rate (μ_max_) predicted for the (*Vp-tdh*) strain was higher (*p* < 0.05) than that for the (*Vp-tlh*) strain; the generation time (*G*) for the (*Vp-tdh*) strain was shorter (*p* < 0.05) than that for the (*Vp-tlh*) strain.

There are few predictive models for viability in live oysters. Reports show that *V. parahaemolyticus* pathogenic strains have longer lag times than the lag times of nonpathogenic strains. An average *R*^2^ value of 0.85 for the Baranyi model was fitted to four kinetic growth profiles of *V. parahaemolyticus* that were inoculated in live Pacific oysters (*C. gigas*). The inactivation rates were −0.006 and −0.004 log CFU/h at 3.6 and 6.2 °C, respectively [31]. In our study, the average *R*^2^ (0.8300 and 0.8100) and *G* values for the (*Vp-tlh*) and (*Vp-tdh*) strains (0.03 and 0.02 h, respectively) were based on naturally contaminated live oysters. These different growth rate values may be due to the growth of *V. parahaemolyticus* inoculated in live oysters, which may differ from the growth of naturally occurring *V. parahaemolyticus* in live oysters. Thus, inoculation might affect the distribution of *V. parahaemolyticus* in oyster tissues. In live oysters, *V. parahaemolyticus* appears to accumulate at higher densities in the digestive glands, gills, visceral mass, and adductor muscle. Moreover, in addition to temperature effects, *V. parahaemolyticus* growth may be influenced by other factors, such as types and levels of competitive flora present among different oyster-growing regions, host defense systems, and the probable release of antimicrobial peptides [32]. Likewise, another developed fitted model had an *R*^2^ of 0.77 and a growth rate of −0.0012; the lag phase and μ_max_ were not determined as *V. parahaemolyticus* decreased to >10 CFU/g in oysters stored at 5 and 10 °C, suggesting that temperatures at or below 10 °C are effective for preventing its growth in shellstock oysters [33]. However, the minimum growth temperature of *V. parahaemolyticus* in oysters has been reported to be 3°C [12]. Similarly, no increase in (*Vp-tlh*) counts in eastern oysters (*C. virginica*) that were harvested from Chesapeake Bay, Maryland for 10 days of storage at 5 and 10 °C was reported [34]. However, our previous study on eastern oysters (*C. virginica*) stored at 7 °C showed that the density of nonpathogenic (*Vp-tlh*) increased (*p* < 0.05) from 1.134 (0 h) to 2.764 log NMP/g at 72 h, while the densities of pathogenic strain (*Vp-tdh*) increased (*p* < 0.05) from −0.824 to 0.519 log NMP/g. Nevertheless, no pathogenic strains were detected (−0.824 log_10_ NMP/g) at 144 h [11]. In this context, although there are few predictive models for the survival of *V. parahaemolyticus* in oysters, the FDA has denoted the lack of information on the postharvest survival at low temperatures.

### 3.2. Effect of Cold Shock on Growth Kinetics of Nonpathogenic (Vp-tlh) and Pathogenic (Vp-tdh) V. Parahaemolyticus In Vitro

**Figure 2:**
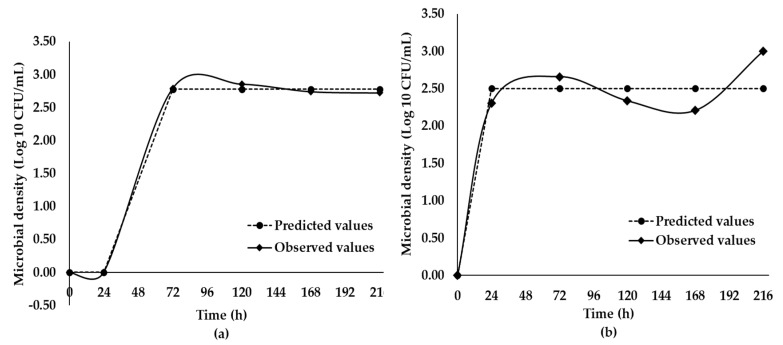
Comparison of the predicted and observed nonpathogenic *tlh*+ (**a**) and pathogenic *tlh+/tdh*+ (**b**) *V. parahaemolyticus* strain growth in tryptone soy broth during refrigerated storage at 7 ± 1 °C for 216 h (9 days). The strains were isolated from live oysters (*Crassostrea virginica*) at 0 h. Predicted growth values were fitted using Equation (1).

The growth curves of (*Vp*-*tlh*) and (*Vp-tdh*) *V. parahaemolyticus* levels were fit to the experimental data and are presented in Figure 2a,b, respectively. These figures show the fit of Equation (1) to the observed data. The initial viable levels of (*Vp*-*tlh*) and (*Vp-tdh*) *V. parahaemolyticu* were equal (3.00 log_10_ CFU/mL). After 24 h, no increase in the (*Vp-tlh*) levels was observed, but the (*Vp-tdh*) levels increased (*p <* 0.05) by 2 log units. The (*Vp*-*tdh*) levels increased significantly (*p* < 0.05) at 168 at 216 h of storage. However, at 216 h, no significant differences (*p* > 0.05) were observed between the log counts of the (*Vp-tlh*) and (*Vp*-*tdh*) strains.

The values for the kinetic growth parameters and the performance statistics of the modified Gompertz model at 7 °C are shown in Table 2. The average *R*^2^ value of the model for the growth curves in broth was 0.9990 for the (*Vp-tlh*) strains and 0.9244 for the (*Vp*-*tdh*) strains and fit the *V. parahaemolyticus* growth; this outcome indicates that the model accurately described *V. parahaemlyticus* growth. Predicted lag time values (λ) of the (*Vp-tdh*) and the (*Vp-tlh*) strains indicated the faster growth and better adaptation of the (*Vp-tdh*) strains to the tested temperature. The maximum specific growth rate (μ_max_) predicted for the (*Vp-tdh*) strains was two times faster (*p* < 0.05) than that for the (*Vp-tlh*) strains, and the generation time (*G*) for the (*Vp-tdh*) strain was shorter (*p* < 0.05) than for the (*Vp-tlh*) strains.

This effect was not expected since metabolism is affected, especially at cold temperatures, and reduced virulence was predictable. However, these results could be explained from those of Tang et al. [35] who observed that the most up-regulated proteins in *V. parahaemolyticus* F8-4 strain, isolated from the American Pacific oyster *Concha Ostreae* and subjected to cold stress at 4 °C for 18 h, were functionally categorized as nucleotide transport and metabolism, transcription, and defense mechanism, indicating that these proteins may play an important role under cold stress. In contrast, another study showed that the nonpathogenic (*Vp-tlh*) strain grew faster than the (*Vp-trh*) strain isolated from raw Korean oysters, regardless of the model medium (in broth and in oyster slurry) at 10 °C; this outcome occurred because the lag phases of the nonpathogenic (*tlh*+) (24.6 h) and pathogenic (*trh*+) (38.7 h) strains were different (*p* < 0.05). In addition, neither the pathogenic nor the nonpathogenic *V. parahaemolyticus* grew at 5 °C, and the concentration of the pathogenic (*trh*+) strain decreased faster than that of the nonpathogenic (*tlh*+) strain [10]. Similarly, it has been reported that pathogenic *(tdh+*) loads decreased by 2.880 and 1.490 log CFU/mL in TSB during storage at 5 °C and 8 °C, respectively, for 10 days [36].

In our study, the differences in *V. parahaemolyticus* growth (Table 2) can be attributed to the intrinsic characteristics of the strains, inoculum cell concentration, and/or to the variation in strain susceptibility to isolation media [37]. These findings indicate that growth of both *V. parahaemolyticus* strains was affected after cold shock and by refrigerated storage. However, the pathogenic and nonpathogenic *V. parahaemolyticus* strains grew at 7 °C, although these strains were isolated from oysters harvested from tropical waters during the winter season. The predicted generation time and lag time values of the (*Vp-tdh*) strains in live oysters and in vitro suggest that the growth characteristics of *V. parahaemolyticus* might vary by strain and storage conditions, such as food matrix and nutrient concentrations, and the opportunity for the cells to repair the damage. As previously mentioned, proliferation and interaction with certain bacterial groups and the oyster defense system may contribute to fluctuations in *V. parahaemolyticus* growth in live oysters [12].

The predicted lag time (λ) values of the (*Vp-tdh*) strains in oyster samples harvested during the winter season may suggest that the upregulation of a virulence mechanism at a cooler temperature may imply a temperature-dependent regulation of virulence gene expression and the utilization of these physiological responses to survive, as previously suggested [38]. The differences in the regulated genes between the strains may be due to a physiological response against environmental stressors. Environmental triggers can disrupt a variety of cell processes and can promote the development of more stress-resistant cells, modulating the fitness and virulence of bacterial pathogens. In this regard, our results confirm our previous findings, where the *tdh* gene was found across the winter with a mean pathogenic (*Vp-tdh*) density in oysters (*C. virginica*) at 1.33 log MPN/g [5]. The behavior of the (*Vp-tdh*) strains at a low storage temperature suggests adaptation to cold stress, which originates from physicochemical changes in the cell structure that favor acclimatization [39]. Several biochemical changes take place during the lag period, such as an increase in the fluidity of the cell membrane due to fatty acid desaturation and the arrest of nucleic acid synthesis [40]. Under these adaptive changes, *V. parahaemolyticus* may express genes that regulate the induction of virulence genes and those involved in other processes, such as the stress response *rpoS* gene and the cold shock *cspA* gene. These changes in the genetic structure of *V. parahaemolyticus* favor adaptation and survival at low temperatures, representing a health hazard [41].

### 3.3. Effect of Cold Shock on Gene Expression of Nonpathogenic (Vp-tlh) and Pathogenic (Vp-tdh) V. Parahaemolyticus In Vitro

To eliminate the natural *Vibrio vulnificus* cells that were present, Limthammahisorn et al. [42] depurated oysters with autoclaved seawater at 25 °C prior to the inoculation of clinical and environmental *V. vulnificus* strains to determine the response to cold shock. Their results indicated that the *V. vulnificus* response to suboptimal temperatures in oysters was similar to that under in vitro conditions. Considering this finding, we used purified nonpathogenic (*Vp-tlh*) and pathogenic (*Vp-tdh*) strains isolated from the live shellstock oysters for RT-PCR amplification in vitro due to the high endogenous bacterial loads found in live shellstock oysters harvested from Veracruz lagoons. We have previously reported that the fecal coliform, *E. coli*, and *V. cholerae* non-O1/nonO139 levels do not decrease to zero in live shellstock oysters (*C. virginica*), even with artificial seawater or ozonated seawater depuration [43]. Therefore, to determine the relationship of the expression of the *rpos* and *cspA* genes on the survival of purified (*Vp-tlh*) and (*Vp-tdh*) strains, the expression of both genes was monitored in vitro during the storage period at 7 °C.

**Figure 3:**
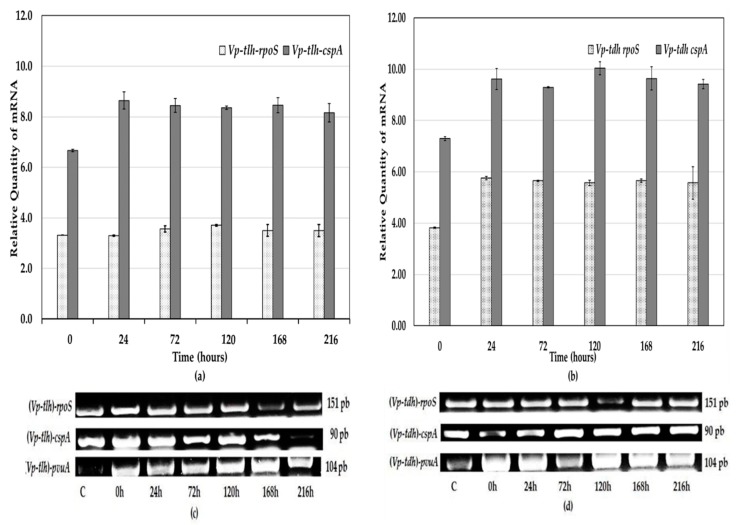
Gene expression analysis of the *rpos* and *CspA* genes of the *V. parahaemolyticus* pathogenic (*Vp-tdh*) and nonpathogenic (*Vp-tlh*) strains isolated from live tropical oysters during refrigerated storage at 7 ± 1 °C for 216 h in tryptone soy broth. The error bars are the standard deviations from three independent experiments. The relative expression levels were calculated with respect to the expression of *pvuA*, which was used as an endogenous reference gene. Reverse transcription PCR (RT-PCR) of the expressed *rpos* and *CspA* genes of the *V. parahaemolyticus* nonpathogenic (*Vp-tlh*) (**a**) and pathogenic (*Vp-tdh*) (**b**) strains was performed. A comparison of the gene expression of *rpos* and *cspA* by an agarose gel electrophoresis analysis of the RT-PCR amplification products of the *V. parahaemolyticus* nonpathogenic (*Vp-tlh*) (**c**) and pathogenic (*Vp-tdh*) (**d**) strains is shown. The cells were exposed to cold shock by decreasing the temperature from 35 °C for 2 h (lane 0 h) to 7 °C for 216 h. The expression levels of *rpos* and *cspA* at 40 cycles of cDNA amplification are shown. The gene expressions levels of *rpos* and (151 bp) and *cspA* (90 bp) showed variations in the band intensity during refrigerated storage for 216 h. The housekeeping gene (*pvuA*) of the 104 bp product was used as an internal control. *V. parahaemolyticus* DNA was used as a positive control (C) for PCR. All experiments were repeated three times.

Figure 3a,b show the average expression of the *rpos* and *cspA* genes from the *V. parahaemolyticus* strains for 216 h of storage at 7 °C. Figure 3a shows that the average relative expression of the *rpoS* gene of the (*Vp-tlh)* strains on day 0 decreased (*p* > 0.05) after 24 h. However, the *rpoS* gene transcript levels at 0 h were not significantly different (*p* > 0.05) from those observed at 72, 120, 168, and 216 h. *rpoS* gene expression was repressed by −0.024-fold after 24 h. Meanwhile, the relative *cspA* gene expression at 0 h increased significantly (*p* < 0.05) after 24 h, remaining relatively constant (*p* > 0.05) during the storage period. *cspA* gene expression was upregulated by 1.9-fold at 24 h. *rpoS* gene expression correlated with the nonpathogenic (*Vp-tlh*) strain levels (*r* = 0.689, *p* = 0.013). However, there was no correlation between the (*Vp-tlh)* strain growth and the *cspA* gene expression (*r* = 0.481, *p =* 0.114) and between the *cspA* and *rpoS* gene expression levels of the (*Vp-tlh*) strain (*r* = 0.294, *p =* 0.353). These results may reflect an increase in the expression of genes that were poorly expressed prior to cold shock, the occurrence of a mixed population of cells in several physiological stages, or a combination of these effects. Figure 3b shows that the expression fold change of the (*Vp*-*tdh*) *rpoS* gene at 0 h increased (*p* < 0.05) at 24 h and remained relatively constant throughout the storage period (stat phase) (*p* > 0.05).

This increase in the expression levels shows that the *rpoS* gene was upregulated by 1.9-fold when the (*Vp*-*tdh*) strain was exposed to a low temperature (7 °C) for 24 h. At the same time, the results showed that the low temperature significantly increased (*p* < 0.05) the expression level of the *cspA* gene at 24 h, which remained relatively constant (*p* > 0.05) until 216 h. mRNA synthesis was induced and after 24 h of cold shock, the *cspA* gene was upregulated by 2.3-fold compared to the 0 h expression level. This (*Vp-tdh*) strain grew faster, showing a shorter lag phase (0.89 h) than the lag phase of the (*Vp-tlh*) strain (37.65 h).

These results contrast with those of Tang et al. [35], who observed that the expression level of *cspA* decreased and the quantity of CspA was 0.12-fold compared with the control in the *V. parahaemolyticus* F8-4 strain that was subjected to cold stress at 4 °C for 18 h. Studies have reported a lag period of approximately 4 h before cell growth is resumed when an exponentially growing culture of *E. coli* is cooled from 37 to 10 °C. The downshift in temperature causes a transient inhibition of most protein synthesis, resulting in a growth lag called the acclimation phase. Cold shock proteins are induced during this lag period, which are essential for the cells to resume growth [44]. The *cspA* gene, a class I *csp* gene, decodes the cold shock protein CspA, which is considered an RNA chaperone that accumulates during growth at low temperatures and modulates both the transcription and translation of the target genes required for low-temperature bacterial survival [45]. In our in vitro assay, we observed that the (*Vp*-*tdh*) strain growth strongly correlated with the expression of the *rpoS* gene (0.97, *p* = 0.00) and the expression of the *cspA* gene (*r* = 0.88, *p* = 0.00). The expression of the *rpoS* and the *cspA* genes correlated (*r* = 0.94, *p* = 0.00) as well. It has been suggested that the *rpoS* gene, a general stress regulator, is the most crucial sigma factor for survival under various stress conditions including the stationary phase [46]. The *rpoS* gene contributes to the management of common factors during bacterial adaptation, including adhesion factors and the excretion of extracellular enzymes, such as lipases and proteases, when the bacterium is dealing with extreme conditions in the surrounding environment. The regulation of gene expression is a common adaptive phenomenon that is observed in bacteria following exposure to environmental stress [46]. It has been shown that the expression ratio of *rpoS* (VP2553) of *V. parahaemolyticus* in the stat phase compared with that in the lag phase was a fold change of 1.65- log_2_ (adjusted *p* < 0.05). Furthermore, the *rpoS* gene can mediate virulence either directly by controlling the expression of virulence factors or indirectly by stimulating a quick adaptation response to improve *V. parahaemolyticus* survival [29].

A cold-adaptation response was observed as both *V. parahaemolyticus* strains adapted and were able to increase their numbers at 7 °C in both live oysters and in vitro assays. However, the relative expression of the *rpoS* and *cspA* genes in the (*Vp-tlh*) strain in vitro were less upregulated compared with the relative gene expressions in the (*Vp-tdh*) strain. As shown in Figure 3c,d, the mRNA transcripts of the *rpoS*, *cspA*, and *pvuA* genes were detected and the levels remained relatively constant despite cold shock. The expression levels of these genes were detected and differentially expressed at all time points as abundance and intensity variations of the transcripts were observed between the (*Vp-tdh*) and (*Vp-tlh*) strains, with the transcripts of the (*Vp-tdh*) *rpoS* genes being more highly expressed than those of the (*Vp-tlh*) strain. Furthermore, the gene expression fold differences between the pathogenic and nonpathogenic strains were observed, e.g., the change in transcription between the (*Vp-tdh*) and (*Vp-tlh*) strains for *rpoS* and *cspA* was 3.2 and 1.2, respectively. The *rpoS* gene provides protection against extreme environments and reinforces the strain’s ability to adapt by linking to the expression of other genes; this protection and adaptation promote survival and a faster growth rate after acclimation to low temperatures, depending on the pathogenicity [46].

A few previous studies have reported the expression of *V. parahaemolyticus* virulence genes under cold stress. The cold-shock responding gene *cpsA* and the global regulator *rpoS* of a clinical strain *V. parahaemolyticus* RIMD2210633 O3:K6 positive showed significant upregulation (4.06 and 3.5-fold change, respectively) at 4 °C, although the expression of *tdh* was activated (2.1-fold change) (*p* < 0.05) at 15 °C [47]. In contrast, the transcription levels of *tdh* in the clinical strain *V. parahaemolyticus* ATCC33847 were upregulated (*p* < 0.05) in shrimp samples and in seawater samples at 9 °C. Additionally, low temperature had a positive effect (*p* < 0.05) on gene expression, including *trh* in the clinical strain ATCC17802 cultured in shrimp and seawater samples and the clinical strain VP2 in seawater samples. However, *tdh* from the clinical strain VP1 that was cultured in shrimp samples demonstrated a lower expression level at 9°C, indicating that the gene expression at a low temperature was irregular among different strains and matrices [48]. Bacterial Csps are vastly conserved small multifunctional nucleic acid-binding proteins that mediate a wide range of physiological functions, including regulation of growth under both normal and cold conditions, stress resistance, and virulence-associated responses, by modulating transcription, translation, and mRNA stability [49]. Csps have been identified as major determinants of pathogenicity in several foodborne disease-causing bacteria, such as *Listeria monocytogenes* [49], *Staphylococcus aureus* [50], and *Brucella melitensis* [51]. Our study showed the *V. parahemolyticus* growth upon cold shock and the induction of *cspA* gene, which suggest, respectively, cellular adaptive mechanisms and an altered metabolic recovery. The (*Vp-tdh*) strain exhibited a shorter lag phase in live oysters (0.33 h) than that in vitro (0.89 h) and the (*Vp-tdh*) strain *cspA* gene was upregulated (2.3-fold) after the cold shock in vitro as well. Thus, it appears that the expression of the *cspA* gene became involved in cold shock response in vitro and it may be implicated in the cold shock response in vivo. There is evidence that *V. vulnificus* survival and tolerance at cold temperature could be due to the expression of cold adaptive genes as the *csp* genes, encoding for putative cold shock proteins, which were differentially expressed in response to in vivo cold shock. Furthermore, in that study the *V. vulnificus* response to cold shock (15 to 4 °C) in oysters was similar to the response in vitro conditions [42].

The current guidelines of the Guide for the Control of Molluscan Shellfish [52] consider testing isolates for the *tdh* gene with a limit of less than 5 *tdh*+ cell-forming units (CFU/g) from shellfish. The guidelines for storing and shipping shellfish that are intended for raw consumption indicate that shellfish need to be brought down to 15.5 °C or less to slow the bacterial growth. In accordance with Mexican Norm NOM-242-SSA1-2009, shellstock oysters should be kept alive and adequately refrigerated at 7 °C for no more than 7 days with a limit of less than 10^4^ MPN/g. However, according to our results, the *V. parahaemolyticus* (*Vp-tdh*) strain had a shorter lag phase and faster growth rate after acclimation to low temperature (7 °C) in live oysters and in vitro. In the context of health risk, further research is needed to better understand the mechanism of *V. parahaemolyticus* survival at low temperatures and its response to cold stress. 

## 4. Conclusions

In the present study, the modified Gompertz model (Equation (1)) produced a good fit to the data for growth of *V. parahaemolyticus tlh*+ and *tlh*+/*tdh*+ on oysters (*R*^2^ = 0.8300, 0.8100, respectively) and in broth (*R*^2^ = 0.9990, 0.9244, respectively) when stored at 7 °C. The predicted lag time (λ), the maximum specific growth rate (μ_max_), and the generation time (G) values indicated a faster growth rate for both strains in live oysters than the growth rate in vitro. The in vitro growth kinetics of the pathogenic (*Vp-tdh*) strain was found to be faster than that of the nonpathogenic (*Vp-tlh*) strain. The in vitro lag phase of the pathogenic strain was shorter than that of the nonpathogenic strain, and its levels increased significantly at 216 h of storage. When *V. parahaemolyticus* (*Vp-tlh*) and (*Vp-tdh*) strains were exposed to the downshift in temperature from 35 °C to 7 °C, they underwent cold stress, which prompted cold shock responses. Our findings suggest that the *V. parahaemolyticus* (*Vp-tlh*) *cspA* cold shock gene and the (*Vp-tdh*) *cspA* and stress *rpoS* genes were inducible in vitro, and an upregulation that was induced by cold shock was observed over the storage time at 7 °C. No induction levels were detected in the *V. parahaemolyticus* (*Vp-tlh*) *rpoS* gene, which seemed to be slightly repressed. The change in the transcription of *rpoS* and *cspA* genes was higher in the *V. parahaemolyticus* (*Vp-tdh*) strain than that in the nonpathogenic (*Vp-tlh*) strain.

Considering these findings, our results indicate that the *V. parahaemolyticus* strains from live tropical shellstock oysters have adaptive tolerance responses to survive and grow at low temperatures and have temperature-dependent expression changes, according to their virulence. Therefore, more research for assessing the growth behavior of *V. parahaemolyticus* at 7 °C in oysters and other seafoods is needed. These phenomena may be of significant importance regarding food safety since cooling regimes do exist in the food industry, which could potentially induce a cold shock phenomenon. Therefore, the authors suggest that storage and transportation temperatures of oysters intended for raw consumption should be at 3 °C instead of 7 °C to control *V. parahaemolyticus* growth. This should be considered in HACCP plans used by the oyster industry to control the risk of *V. parahaemolyticus* to human health. Further research should be undertaken to understand how the molecular mechanisms of the cold shock response may promote the survival and gene expression changes in pathogenic *V. parahaemolyticus*, which naturally contaminates live oysters, to identify the best food preservation process under low temperatures to improve seafood safety.

## Figures and Tables

**Table 1:** Primers used for molecular analysis of nonpathogenic and pathogenic genes and specific primers used for qPCR amplification of housekeeping genes of *Vibrio parahaemolyticus*.

Gene (ID)		Sequence (5′-3′)	T*_m_* (°C)	References
*tlh* (VPA0226)	tl-f	AAA GCG GAT TAT GCA GAA GCA CTG	58 ^a^	[3]
	tl-r	GCT ACT TTC TAG CAT TTT CTC TGC		
*tdh* (GU971653) *	L-tdh	GTA AAG GTC TCT GAC TTT TGG AC	58 ^a^	[3]
	R-tdh	TGG AAT AGA ACC TTC ATC TTC ACC		
*trh* (GU971654) *	trh-f	TTG GCT TCG ATA TTT TCA GTA TCT	58 ^a^	[3]
	trh-r	CAT AAC AAA CAT ATG CCC ATT TCC G		
*orf8* (AP00581) *	F-O3MM824	AGG ACG CAG TTA CGC TTG ATG	60 ^b^	[26]
	R-O3MM1192	CTA ACG CAT TGT CCC TTT GTA G		
*16S rRNA*	F-10-30:	GAGTTTGATCMTGGCTCAG	F	[22,23]
	R-1492:	GGTTACCTTGTTACGACTT		
*rpoS* (VP2553)	Fw-	GACAATGCGTCAGAGACG	F	[27]
	Rv-	GAGGTGAGAAGCCAATTTC		
*cspA* (VPA1289)	F-	TATCGTTGCTGACGGTTTCA	F	[28]
	R-	TCAGTCGCTTGAGGACCTTT		
*pvuA* VPA1656)	156F-1	CAAACTCACTCAGACTCCA	F	[29]
	56R-	CGAACCGATTCAACACG		

Positive strains: * Accession number; ^a^ CAIM 1772; ^b^ CAIM 1400.

**Table 2:** Parameter values using the modified Gompertz model for *V. parahaemolyticus* (*tlh*+ and *tlh+/tdh*+) growth at 7 °C during 168 h in vivo (oyster) and 216 h in vitro.

*V. parahaemolyticus*	μ_max_ (h^−^^1^)	λ (h)	*A*	*G* (h)	*R* ^2^	*S_yx_*
**In vivo (oyster)**						
*tlh*	8.68	0.39 (23.4 min)	0.18	0.08 (4.8 min)	0.8300	0.0001
*tdh*	28.63	0.33 (19.8 min)	0.62	0.02 (1.2 min)	0.8100	0.0001
**In vitro**						
*tlh*	2.32	37.65 (2,259.0 min)	2.78	0.30 (18.0 min)	0.9990	0.0422
*tdh*	5.29	0.89 (53.4 min)	2.50	0.13 (7.8 min)	0.9244	0.2664

μ_max_ = maximum specific growth rate; λ = lag time; *A =* observed maximum growth index of population density to stationary phase; *G* = generation time.

## References

[1] Wang R., Zhong Y., Gu X., Yuan J., Saeed A., Wang S. (2015). The pathogenesis, detection, and prevention of Vibrio parahaemolyticus. Front. Microbiol..

[2] Mala W., Alam A., Angkititrakul S., Wongwajana S., Lulitanond V., Huttayananont S., Chomvarin C. (2016). Serogroup, virulence, and molecular traits of Vibrio parahaemolyticus isolated from clinical and cockle sources in northeastern Thailand. Infect. Genet. Evol..

[3] Bej A.K., Patterson D.P., Brasher C., Vickery M.C.L., Jones D., Kaysner C. (1999). Detection of total and hemolysin producing Vibrio parahaemolyticus in shellfish using multiplex PCR amplification of tlh, tdh and trh. J. Microbiol. Methods.

[4] Raghunath P. (2015). Roles of thermostable direct hemolysin (TDH) and TDH-related hemolysin (TRH) in Vibrio parahaemolyticus. Front. Microbiol..

[5] López K., Pardío V., Lizárraga L., Williams J., Martínez D., Flores A., Uscanga R., Rendón K. (2015). Environmental parameters influence on the dynamics of total and pathogenic Vibrio parahaemolyticus densities in Crassostrea virginica harvested from Mexico’s Gulf coast. Mar. Pollut. Bull..

[6] Hernández-Díaz L., León-Sicairos N., Velázquez-Román J., Flores-Villaseñor H., Guadrón-Lanos A., Martínez-García J., Vidal J., Canizalez-Román A. (2015). A pandemic Vibrio parahaemolyticus O3:K6 clone causing most associated diarrhea cases in the Pacific Northwest coast of Mexico. Front. Microbiol..

[7] Chao G., Jiao X., Zhou X., Yang Z., Huang J., Pan Z., Zhou L., Qian X. (2009). Serodiversity, pandemic O3:K6 clone, molecular typing and antibiotic susceptibility of foodborne and clinical Vibrio parahaemolyticus isolates in Jiangsu, China. Foodborne Pathog. Dis..

[8] Infomex Veracruz Sistema de Solicitudes de Información del Estado de Veracruz de Ignacio de la Llave. Listado Nominal de Resultados. https://infomexveracruz.org.mx/infomexveracruz/default.aspx.

[9] Aung M.M., Chang Y.S. (2014). Temperature management for the quality assurance of a perishable food supply chain. Food Control.

[10] Yoon K., Min K., Jung Y., Kwon K., Lee J., Oh S. (2008). A model of the effect of temperature on the growth of pathogenic and nonpathogenic Vibrio parahaemolyticus isolated from oysters in Korea. Food Microbiol..

[11] Flores A., Pardío V., López K., Lizárraga L., Uscanga R. (2015). Growth and survival of total para pathogenic Vibrio parahaemolyticus in Americano oyster (Crassostrea virginica) under cold storage. Salud Púb. México.

[12] Gooch J., Depaola A., Owers J., Marshall D. (2002). Growth and survival of Vibrio paraaemolyticus in postharvest American oysters. J. Food Prot..

[13] Horn G., Hofweber R., Kremer W., Kalbitzer H. (2007). Structure and function of bacterial cold shock proteins. Cell. Mol. Life Sci..

[14] Phadtare S., Alsina J., Inouye M. (1999). Cold-shock response and cold-shock proteins. Curr. Opin. Microbiol..

[15] Yang L., Zhou D., Liu X., Han H., Zhan L., Guo Z., Zhang L., Qin C., Wong H., Yang R. (2009). Cold-induced gene expression profiles of Vibrio parahaemolyticus: A time-course analysis. FEMS Microbiol. Lett..

[16] Comisión Nacional de Acuacultura y Pesca (CONAPESCA) Anuario Estadístico de Acuacultura y Pesca 2017. http://www.gob.mx/conapesca/documentos/anuario-estadistico-de-acuacultura-y-pesca.

[17] Lara A., Contreras F., Barba E., Castañeda O., Pérez M., Cruz Angón A. (2011). Lagunas Costeras y Estuarios. La Biodiversidad en Veracruz: Estudio del Estado.

[18] Ramírez Elvira K., López-Hernández K., Pardío Sedas V., Mendoza López M., Flores Primo A., Alarcón Elvira F. Identificación de Vibrio parahaemolyticus en ostiones expendidos en la zona conurbada Veracruz-Boca del Río y Alvarado, Veracruz. Proceedings of the Food Safety Congress 2017.

[19] Pardío V., Wong I., Lizárraga L., López K., Flores A., Barrera G., Alarcón F., Fernández C., Diarte-Plata G. (2018). Survival differences of Vibrio vulnificus and Vibrio parahaemolyticus strains in shellstock oysters (Crassostrea virginica) from harvest to sale: A risk perspective. Molluscs.

[20] Secretaría de Salud Gobierno de México. NOM-242-SSA1-2009. http://portal.salud.gob.mx/.

[21] Secretaría de Economía Gobierno de México. NMX-FF-001-SCFI-2011. http://www.economia.gob.mx/.

[22] Farrelly V., Rainey F., Stackebrandt E. (1995). Effect of genome size and rrn gene copy number on PCR amplification of 16S rRNA genes from a mixture of bacterial species. Infect. Immun..

[23] Turner S., Pryer K., Miao V., Palmer J. (1999). Investigating deep phylogenetic relationships among cyanobacteria and plastids by small subunit rRNA sequence analysis. J. Eukaryot. Microbiol..

[24] Chomczynski P., Sacchi N. (1987). Single-step method of RNA isolation by acid guanidinium thiocyanate phenol chloroform extraction. Anal. Biochem..

[25] Manchester K.L. (1996). Use of UV methods for measurement of protein and nucleic acid concentrations. Biotechniques.

[26] Myers M., Panicker G., Bej A. (2003). PCR detection of a newly emerged pandemic Vibrio parahaemolyticus O3: K6 pathogen in pure cultures and seeded waters from the Gulf of Mexico. Appl. Environ. Microbiol..

[27] Ma Y., Sun X., Xu X., Zhao Y., Pan Y., Hwang C., Wu V. (2015). Investigation of reference genes in Vibrio parahaemolyticus for gene expression analysis using quantitative RT-PCR. PLoS ONE.

[28] Meng L., Alter T., Aho T., Huehn S. (2015). Gene expression profiles of Vibrio parahaemolyticus in viable but non-culturable state. FEMS Microbiol. Ecol..

[29] Coutard F., Lozach S., Pommepuy M., Hervio-Heath D. (2007). Real-time reverse transcription-PCR for transcriptional expression analysis of virulence and housekeeping genes in viable but nonculturable Vibrio parahaemolyticus after recovery of culturability. Appl. Environ. Microbiol..

[30] Schmittgen T., Livak K. (2008). Analyzing real-time PCR data by the comparative C_T_ method. Nat. Protoc..

[31] Fernandez-Piquer J., Bowman J., Tamplin M. (2011). Predictive models for the effect of storage temperature on Vibrio parahaemolyticus viability and counts of total viable bacteria in Pacific oysters (Crassostrea gigas). Appl. Environ. Microbiol..

[32] Cabello A., Espejo R., Romero J. (2005). Tracing Vibrio parahaemolyticus in oysters (Tiostrea chilensis) using a Green Fluorescent Protein tag. J. Exp. Mar. Biol. Ecol..

[33] Parveen S., Dasilva L., DePaola A., Bowers J., White C., Munasinghe K., Brohawn K., Mudoh M., Tamplin M. (2013). Development and validation of a predictive model for the growth of Vibrio parahaemolyticus in post-harvest shellstock oysters. Int. J. Food Microbiol..

[34] Mudoh M., Parveen S., Schwarz J., Rippen T., Chaudhuri A. (2014). The effects of storage temperature on the growth of Vibrio parahaemolyticus and organoleptic properties in oysters. Front. Public Health.

[35] Tang J., Jia J., Chen Y., Huang X., Zhang X., Zhao L., Hu W., Wang C., Lin C., Wu Z. (2018). Proteomic analysis of Vibrio parahaemolyticus under cold stress. Curr. Microbiol..

[36] Burnham V., Janes M., Jakus L., Supan J., DePaola A., Bell J. (2009). Growth and survival differences of Vibrio vulnificus and Vibrio parahaemolyticus strains during cold storage. J. Food Sci..

[37] Hara-Kudo Y., Nishina T., Nakagawa H., Konuma H., Hasegawa J., Kumagai S. (2001). Improved method for detection of Vibrio parahaemolyticus in seafood. Appl. Environ. Microbiol..

[38] Mahoney J., Gerding M., Jones S., Whistler C.A. (2010). Comparison of the pathogenic potentials of environmental and clinical Vibrio parahaemolyticus strains indicates a role for temperature regulation in virulence. Appl. Environ. Microbiol..

[39] Phadtare S., Inouye M. (2004). Genome-wide transcriptional analysis of the cold shock response in wild-type and cold-sensitive, quadruple-csp-deletion strains of Escherichia coli. J. Bacteriol..

[40] Phadtare S., Severinov K. (2010). RNA remodeling and gene regulation by cold shock proteins. RNA Biol..

[41] Trevors J., Bej A., Mojib N., van Elsas J., van Overbeek L. (2012). Bacterial gene expression at low temperatures. Extremophiles.

[42] Limthammahisorn S., Brady Y., Arias C. (2009). In vivo gene expression of cold shock and other stress-related genes in Vibrio vulnificus during shellstock temperature control conditions in oysters. J. Appl. Microbiol..

[43] López Hernández K., Pardío Sedas V., Rodríguez Dehaibes S., Suárez Valencia V., Rivas Mozo I., Martínez Herrera D., Flores Primo A., Uscanga Serrano R. (2018). Improved microbial safety of direct ozone-depurated shellstock Eastern oysters (Crassostrea virginica) by superchilled storage. Front. Microbiol..

[44] Jiang W., Hou Y., Inouye M. (1997). CspA, the major cold-shock protein of Escherichia coli, is an RNA chaperone. J. Biol. Chem..

[45] Michaux C., Holmqvis E., Vasicek E., Sharan M., Barquist L., Westermann A., Gunn J., Vogel J. (2017). RNA target profiles direct the discovery of virulence functions for the cold-shock proteins CspC and CspE. Proc. Natl. Acad. Sci. USA.

[46] Dong T., Schellhorn H. (2010). Role of RpoS in virulence of pathogens. Infect. Immun..

[47] Urmersbach S., Aho T., Alter T., Hassan S., Autio R., Huehn S. (2015). Changes in global gene expression of Vibrio parahaemolyticus induced by cold- and heat-stress. BMC Microbiol..

[48] Zhao A., Liu H., Sun W., Li Q., Pan Y., Zhao Y. (2015). Irregular virulence genes expression of Vibrio parahaemolyticus in shrimp or seawater matrix. J. Microbiol. Biotechnol..

[49] Eshwar A., Guldimann C., Oevermann A., Tasara T. (2017). Cold-shock domain family proteins (Csps) are involved in regulation of virulence, cellular aggregation, and flagella-based motility in Listeria monocytogenes. Front. Cell Infect. Microbiol..

[50] Sahukhal G.S., Elasri M. (2014). Identification and characterization of an operon, msaABCR, that controls virulence and biofilm development in Staphylococcus aureus. BMC Microbiol..

[51] Wang Z., Wang S., Wu Q. (2014). Cold shock protein A plays an important role in the stress adaptation and virulence of Brucella melitensis. FEMS Microbiol. Lett..

[52] U.S. Food and Drug Administration National Shellfish Sanitation Program Guide for the Control of Molluscan Shellfish: 2017 Revision. http://www.fda.gov/Food/GuidanceRegulation/FederalStateFoodPrograms/ucm2006754.htm.

